# Associations between Cholesterol Intake, Food Sources and Cardiovascular Disease in Chinese Residents

**DOI:** 10.3390/nu16050716

**Published:** 2024-02-29

**Authors:** Yuxue Cao, Yan Yu

**Affiliations:** Department of Nutrition and Food Safety, School of Public Health, Xi’an Jiaotong University Health Science Center, Xi’an 710061, China; usk_re@stu.xjtu.edu.cn

**Keywords:** cholesterol intake, food sources, cardiovascular disease, Chinese residents, egg intake, CHNS

## Abstract

Cholesterol is a nutrient commonly found in the human diet. The relationship between dietary cholesterol, its sources, and cardiovascular disease (CVD) is still a topic of debate. This study aimed to investigate the association between dietary cholesterol, its sources, and cardiovascular events in a Chinese population. The present study analyzed data from the China Health and Nutrition Survey (CHNS) cohort between 1991 and 2015. This study analyzed data from 3903 participants who were 40 years of age or older at baseline and had no history of cardiovascular disease, diabetes, or hypertension. During a median follow-up of 14 years, 503 cardiovascular disease events were identified through follow-up questionnaires administered every 2–3 years. The events included fatal and nonfatal coronary heart disease, stroke, heart failure, and other cardiovascular disease deaths. Cox regression was used to estimate risk ratios (HR) for CVD events after adjusting for demographic, socioeconomic, and behavioral factors. It was discovered that sources of dietary cholesterol varied among different subgroups of the population. The top three sources of cholesterol among all participants were eggs, red meat, and seafood, accounting for 57.4%, 28.2%, and 9.0% of total daily cholesterol intake, respectively. The present study found that there was a significant association between total dietary cholesterol intake, and the risk of developing cardiovascular disease (adjusted HR [95% CI]: total cholesterol (highest and lowest quartiles compared) 1.57 [1.17–2.11]). Cholesterol from poultry, seafood, and eggs was also significantly associated with a reduced risk of CVD (adjusted HR [95% CI]: poultry 0.18 [0.04–0.82], seafood 0.11 [0.02–0.54], and eggs 0.16 [0.03–0.73]). After adjusting for daily caloric intake, daily fat intake, and daily saturated fat intake, the previously observed association between red meat cholesterol and cardiovascular events (unadjusted HR [95% CI]: 0.44 [0.35–0.55]) was no longer statistically significant (adjusted HR [95% CI]: 0.21 [0.04–1.01]).

## 1. Introduction

Cardiovascular disease (CVD) is a significant health concern that has a major impact on society. Extensive research has been conducted to examine various diet-related risk factors. The hypothesis that dietary cholesterol contributes to the risk of heart disease was first proposed in 1968 based on the research literature available at that time [1,2]. Previous studies have demonstrated that cholesterol biosynthesis is directly regulated by cholesterol levels in the body [3]. However, the response of plasma cholesterol to dietary cholesterol varies significantly among animal species, within animal species, and between individuals [4]. Therefore, the association between dietary cholesterol and the risk of CVD remains uncertain. Although the United States and China eliminated dietary cholesterol limits for their populations around 2015 [5,6], recent findings have led to calls for reconsideration of these limits [7]. A pooled analysis in the U.S. that included 29,615 participants from six cohorts found a linear association between increased dietary cholesterol intake and the risk of cardiovascular disease and all-cause mortality [8]. A meta-analysis of 27,078 men who participated in the Alpha-Tocopherol, Beta-Carotene Cancer Prevention (ATBC) study found that an increase of 300 milligrams in daily dietary cholesterol intake was associated with a significant age-adjusted increase in the risk of all-cause, cardiovascular, heart disease, and cancer deaths by 8% to 10% [9]. Several other prospective cohort studies have reported similar findings [10,11,12]. However, there are some conflicting results, especially in Asian populations. The Japanese Adult Health Study found that a high intake of animal fat and cholesterol was significantly associated with a reduced risk of cerebral infarction death [13]. Similarly, a study of 18,914 Chinese adults aged 20 years and older found no association between total dietary cholesterol and total mortality, and cholesterol intake from eggs was negatively associated with total mortality (*p* for trend < 0.001) [14]. An example that showcases the extent of the impact is the group of vegetarians, who consume considerably less dietary cholesterol compared to omnivores. A study conducted in China encompassing 170 Buddhist monks (vegetarians) and 126 omnivores revealed that vegetarians exhibited significantly lower indicators of heart disease, such as total cholesterol, as well as a reduced predictive probability of developing coronary heart disease in comparison to omnivores [15]. Additionally, a meta-analysis comprising 10 prospective cohort studies reported that vegetarians had a notably diminished risk of ischemic heart disease incidence and/or mortality (RR 0.75; 95% CI, 0.68–0.82) [16].

However, the dose–response evidence for the association of dietary cholesterol with CVD in China remains largely inconclusive because of differences in dietary habits between Chinese and Western populations [14,17,18]. Previous studies have found that the average daily intake of dietary cholesterol in Chinese people aged 60 years and older reached 253.9 mg in 2011, which is higher than the average dietary cholesterol intake [19]. In addition, as early as the 2004 survey, the daily intake exceeded the global average in 2010 [20]. Therefore, the aim of this study was to investigate the association of dietary cholesterol and the proportion of its food sources with the risk of CVD in the Chinese population over 40 years of age by analyzing data from a comprehensive prospective cohort study (CHNS).

## 2. Materials and Methods

### 2.1. Study Population

**Figure 1:**
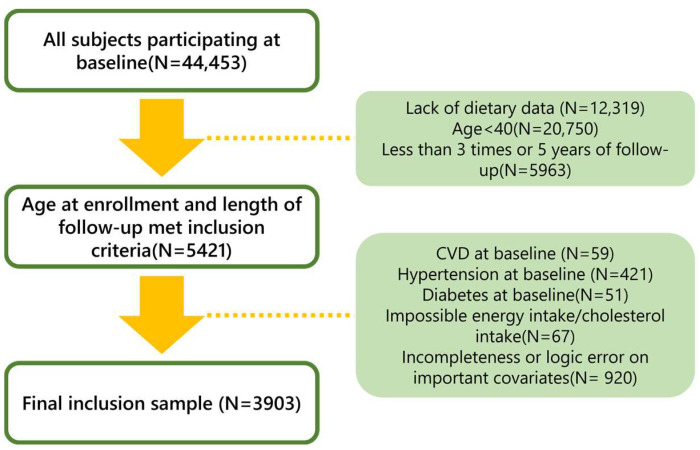
Flow chart of study participants for analysis.

The China Health and Nutrition Survey (CHNS) is a national prospective cohort study that has been recruiting participants since 1989. It follows participants from nine provinces and three autonomous cities every 2–3 years, collecting dietary, anthropometric, and other data from individuals, households, and communities. More information on the CHNS can be found elsewhere [21,22]. For this study, we used eight waves of publicly available survey data (CHNS 1991, 1993, 1997, 2000, 2004, 2006, 2009, and 2011). Because dietary information for 2015 was not available, data were limited to disease and mortality during that period. Study exclusion criteria included individuals under 40 years of age, individuals with 2 or fewer follow-up visits, individuals with implausible high or low energy intake (<500 or >8000 kcal/day) and those with implausible dietary cholesterol intake >2000 mg/day, individuals with missing dietary intake or missing key covariates (e.g., smoking status, history of chronic disease, medication use, and height, weight, and blood pressure measurements), and participants with cardiovascular disease, physician-diagnosed hypertension, and diabetes at baseline [23]. These criteria resulted in a final sample analysis of 3903 participants (Figure 1).

### 2.2. Study Variables

The CHNS was approved by the institutional review boards of the University of North Carolina (Chapel Hill, NC, USA) and the National Institute of Nutrition and Food Safety (Chinese Center for Disease Control and Prevention). Informed consent was obtained from all participants prior to participation. Dietary information was collected by trained interviewers on three consecutive, randomly selected days within a given week at both the individual and household levels [24,25,26]. To report an individual’s dietary intake, participants recorded all foods consumed (including meals and snacks) within the previous 24 h. Interviewers used food models and pictures to record all meal locations, types, and quantities. The average daily intake from each participant’s three-day food recall was used as dietary data for this study.

The study focused on the incidence of total cardiovascular disease (CVD), defined as a composite of angina, stroke, acute myocardial infarction, ischemic heart disease, and pulmonary heart disease. In each follow-up wave, participants were asked to complete a standardized questionnaire asking about physician-diagnosed CVD [27]. The study recorded person-years from the baseline survey to the year of censoring (2015), the incidence of lost visits (including deaths unrelated to cardiac issues, lost visits, or refusals of examination), or a CVD endpoint (illness or death caused by heart disease), whichever came first. During each follow-up wave, participants were asked to complete a standardized questionnaire and report any physician-diagnosed cardiovascular disease. In the event of a subject’s death during the follow-up period, the investigator recorded the cause of death.

Covariates were assessed by trained personnel using standardized questionnaires to collect information on sociodemographic characteristics (age, sex, and geographic location at recruitment), lifestyle factors (smoking), and dietary factors. Biased and figurative language was avoided. Age was calculated from self-reported birthdays at the time of baseline data collection. Uniformly measured height and weight were used to derive body mass index (BMI), defined as weight (kg) divided by the square of height (m^2^). Uniformly measured blood pressure values were used to assess the baseline risk of hypertension for each participant, calculated as the mean of three measurements of systolic and diastolic blood pressure. Participants were categorized into urban and rural residents based on area of permanent residence. Geographically, the Qinling and Huaihe rivers were used to identify the northern and southern regions of China (there are significant differences in living and eating habits between residents of northern and southern China, and these differences are not the same as those between rural and urban residents). Smoking/drinking status was categorized into two groups: nonsmokers/nondrinkers and smokers/drinkers based on self-reported smoking/drinking status.

We used the Chinese Food Composition Table to calculate the individual daily intake of cholesterol for each food item in the dietary data. Animal foods were also categorized into six groups according to the grouping of the composition table: eggs, red meat, seafood (includes fish, shrimp, crabs, shellfish, mollusks, etc., in fresh or marine water), poultry, dairy, and others. Participants were divided into four groups based on quartiles of daily cholesterol intake: Q1 (≤79.0 mg/day), Q2 (≤183.3 mg/day), Q3 (≤362.5 mg/day), and Q4 (>362.5 mg/day). Data whose baseline characteristics did not fit a normal after normality testing are expressed as medians (quartiles). Count data are expressed as N (%). A stratified Cox proportional risk model was used to examine the association between cholesterol intake and the proportion of its food source and the incidence of cardiovascular disease (CVD). Risk ratios (HRs) and 95% confidence intervals (CIs) were calculated with the lowest intake group as the reference. The proportional risk assumption in the adjusted Cox model was checked by evaluating the weighted Schoenfeld residuals [28]. There was no evidence of violation of the proportionality assumption for risk factors other than age (*p* > 0.05), and we stratified for age in all subsequent models.

### 2.3. Statistic

To examine the association between cholesterol intake and CVD, we developed adjusted models and included risk factor covariates associated with known or suspected CVD events. Model 1 was an unadjusted model. Model 2 adjusted for sex, age (stratified), BMI (<18.5, 18.5–23.9, 24–27.9, or 28 kg/m^2^ [29]), alcohol consumption, daily fat intake, daily saturated fat intake, and daily caloric intake. Model 3 further adjusted for risk of hypertension (individuals were considered at risk of hypertension if their mean systolic blood pressure was greater than 140 mm/Hg or diastolic blood pressure was greater than 90 mm/Hg, based on physical measurements taken during the baseline survey), geographic location (north or south), residence (urban or rural), and smoking status (never smoked or smoked).

Several models were developed to examine the association between dietary sources of cholesterol intake and CVD. After a one-way Cox analysis of the four main food sources of cholesterol, the one-way factors were included together in model 1. Model 2 was adjusted for total cholesterol intake. Model 3 was further adjusted for sex, age (stratified), BMI (<18.5, 18.5–23.9, 24–27.9, or 28 kg/m^2^), and total fat intake.

All statistical analyses were performed with R version 4.3.1, provided by the R Foundation for Statistical Computing, Vienna, Austria. The “cox.zph” function in the survival package was used to test the proportional risk hypothesis, and the “coxph” function in the survival package was used to test the hazard ratio hypothesis. The Cox model was fitted using the “coxph” function in the survival package. A two-tailed *p*-value less than 0.05 was considered statistically significant.

## 3. Results

This study included 3903 participants with 55,264 person-years of follow-up data. During a median follow-up of 14.0 years (interquartile range: 9.0–20.0; maximum: 24.0), all 3903 participants experienced 503 incident CVD events. At baseline, the mean (SD) age was 54.5 (10.7) years, and 1868 (47.9%) were men. Table 1 shows the characteristics of the participants based on quartile-stratified levels of dietary cholesterol consumption. The four intake levels of participants differed significantly in terms of sex, BMI, dietary intake, environment, behavior, and incident CVD. The median daily dietary cholesterol consumption was 183.3 (interquartile range, 79.0–362.5) mg. The mean (SD) was 248.3 (214.8) mg per day. The unadjusted incidence of incident CVD was 9.1 per 1000 person-years (95% CI, 8.33–9.94).

**Figure 2:**
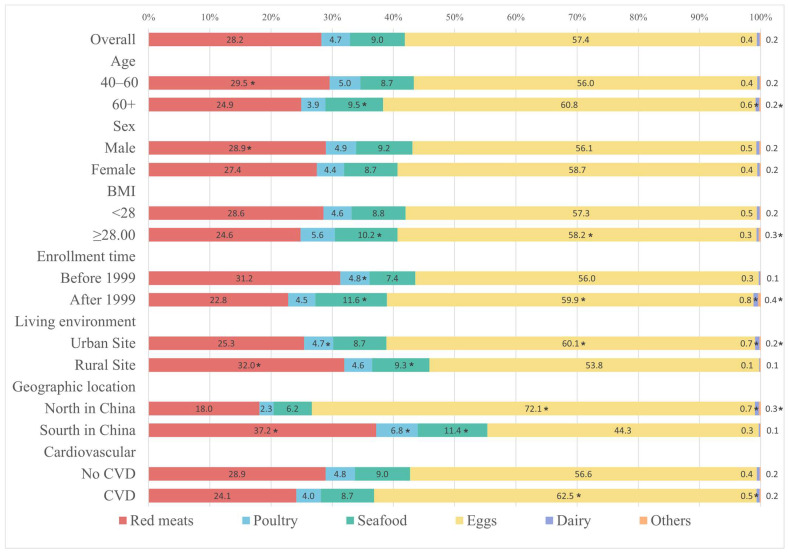
Food sources of dietary cholesterol among the Chinese population over 40 by subgroup. Asterisks indicate the level of statistical significance: * *p* < 0·05.

In all participants, the top three sources of cholesterol were eggs, red meat, and seafood, contributing 57.4%, 28.2%, and 9.0% to total daily cholesterol intake, respectively (Figure 2). The percentage of cholesterol intake from red meat was significantly higher in the 40–60 age group than the over 60 group (29.5% vs. 24.9%, *p* < 0.001), and in males compared to females (28.9% vs. 27.4%, *p* < 0.001). Additionally, the intake of red meat, poultry, seafood, and eggs was higher among rural residents compared to urban residents (red meat: 32.0% vs. 25.3%, *p* < 0.001; poultry: 4.6% vs. 4.7%, *p* < 0.001; seafood: 9.3% vs. 8.7%, *p* < 0.001), and among residents of south China compared to those of north China (red meat: 37.2% vs. 18.0%, *p* < 0.001; poultry: 6.8% vs. 2.3%, *p* < 0.001; seafood: 11.4% vs. 6.2%, *p* = 0.002). Cholesterol intake from eggs differed significantly among all subgroups except for sex and age (higher intake group: BMI ≥ 28.0, 58.2% vs. 57.3; enrollment time after 1999, 59.9% vs. 56.0%; unemployed, 61.5% vs. 53.8%; urban residents, 60.1% vs. 53.8%; north China residents, 72.1% vs. 44.3%; participants with incident CVD, 62.5% vs. 56.6%; all *p* value < 0.001). Cholesterol intake from dairy and other sources was significantly higher among the over 60 group compared to the 40–60 age group (dairy: 0.6% vs. 0.4%, *p* = 0.022), among urban residents compared to rural residents (dairy: 0.7% vs. 0.1%, *p* < 0.001; other: 0.2% vs. 0.1%, *p* < 0.001), and among residents of north China compared to those in the south (dairy: 0.7% vs. 0.3%, *p* < 0.001; other: 0.3% vs. 0.1%, *p* = 0.004).

**Figure 3:**
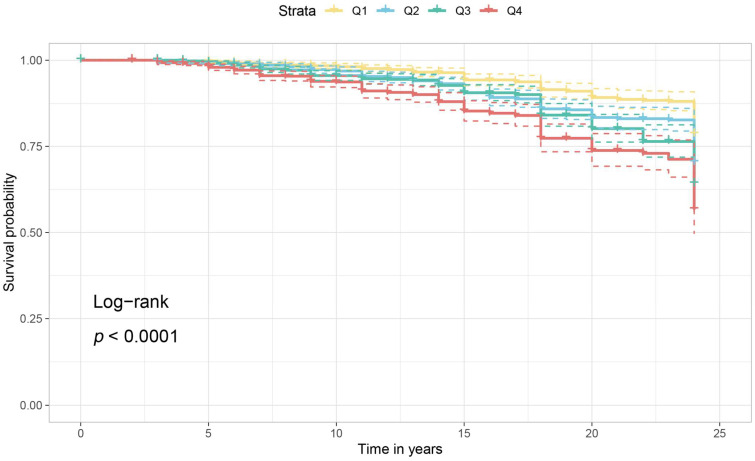
Survival curves for subgroups of total cholesterol intake quartiles. One-way survival curves for total cholesterol intake and incident CVD grouped by quartiles.

**Figure 4:**
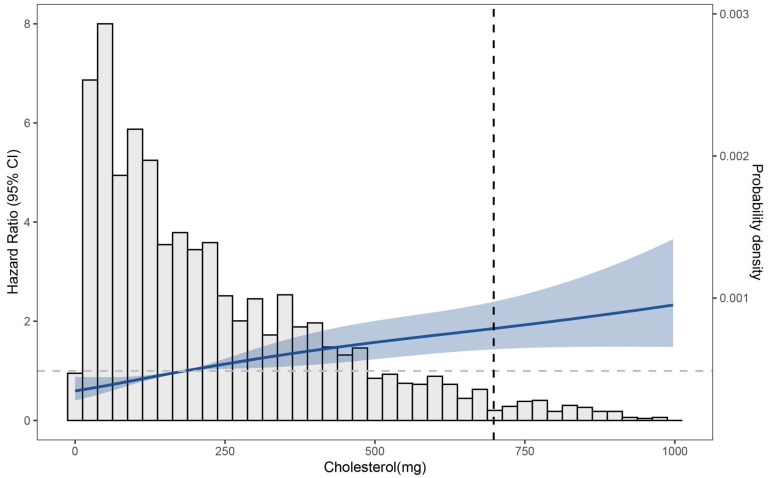
Associations between dietary cholesterol consumption and incident CVD.

**Figure 5:**
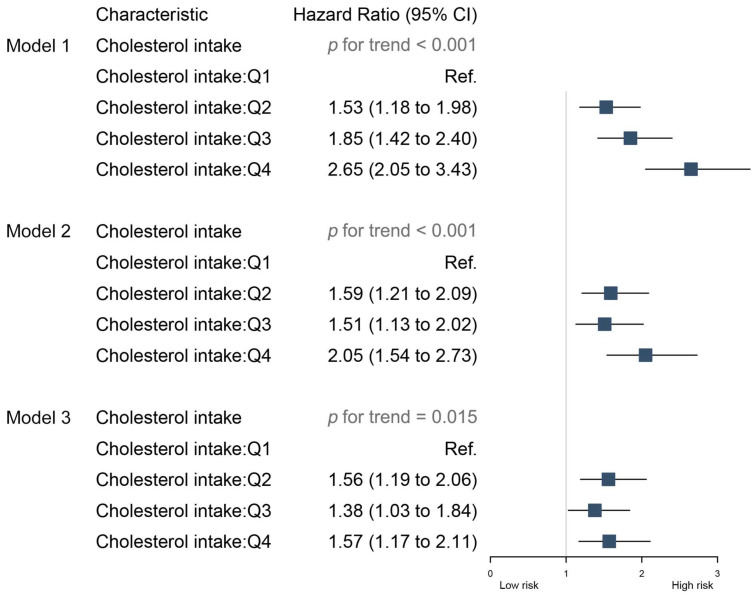
Association between quartile groupings of total cholesterol intake and cardiovascular events. Model 1 was the unadjusted model; model 2 adjusted for age, BMI, sex, alcohol consumption, daily fat intake, daily saturated fat intake, and daily caloric intake; model 3 additionally adjusted for risk of hypertension, residence, geographic location, and smoking status.

In unadjusted model 1 of dietary cholesterol consumption and incident CVD, higher dietary cholesterol consumption was associated with a higher risk of incident CVD (*p* for trend < 0.001) (Figure 3 and Figure 4). The same association was observed in model 2 (adjusted for age, BMI, sex, alcohol consumption, daily fat intake, daily saturated fat intake, and daily caloric intake) (adjusted HR [95% CI]: (highest and lowest quartiles compared) 2.05 [1.54–2.73], *p* for trend < 0.001) and model 3 (additionally adjusted for risk of hypertension, residence, geographic location, and smoking status) (adjusted HR [95% CI]: (highest and lowest quartiles compared) 1.57 [1.17–2.11], *p* for trend = 0.015) (Figure 5).

**Figure 6:**
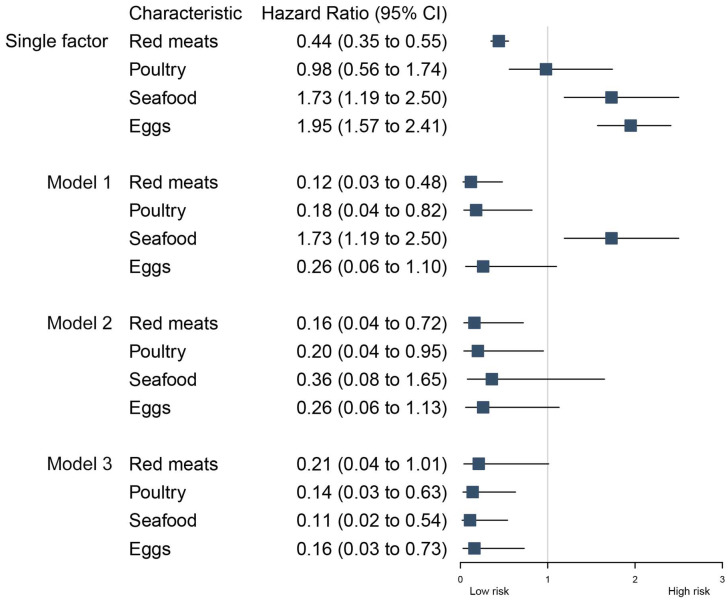
Relationship between percentage of cholesterol from different sources and cardiovascular events. Model 1 was unadjusted model; model 2 adjusted for total cholesterol intake; model 3 further adjusted for daily caloric intake, daily fat intake, and daily saturated fat intake.

The results of the single factor analyses indicate that the intake of cholesterol from red meat sources as a percentage of total cholesterol intake is associated with a lower risk of incident CVD (HR, 0.44 [95% CI, 0.35–0.55]). Conversely, the intake of cholesterol from seafood (HR, 1.73 [95% CI, 1.19–2.50]) and eggs (HR, 1.95 [95% CI, 1.57–2.41]) is associated with a higher risk of incident CVD. In model 1, all single factors were placed in one model. The results showed that cholesterol from red meat (adjusted HR, 0.12 [95% CI, 0.03–0.48]) and poultry (adjusted HR, 0.18 [95% CI, 0.04–0.82]) sources, as a percentage of total cholesterol intake, were connected with a lower risk of incident CVD. However, the associations between cholesterol from egg sources as a percentage of total cholesterol intake and incident CVD (adjusted HR, 0.26 [95% CI, 0.06–1.10]) were no longer significant after adjusting for other sources of cholesterol consumption. The associations between seafood cholesterol as a percentage of total cholesterol intake and incident CVD were no longer significant after adjusting for total dietary cholesterol intake in model 2 (adjusted HR, 0.36 [95% CI, 0.08–1.65]). In model 3, we further adjusted for daily caloric intake, daily fat intake, and daily saturated fat intake. The association between cholesterol derived from red meat and CVD incident was no longer significant (adjusted HR, 0.21 [95% CI, 0.04–1.01]), whereas cholesterol derived from poultry (adjusted HR, 0.14 [95% CI, 0.03–0.63]), seafood (adjusted HR, 0.11 [95% CI, 0.02–0.54]), and eggs (adjusted HR, 0.16 [95% CI, 0.03–0.73]) was significantly associated with a reduced risk of CVD incident (Figure 6).

There were 503 incident cardiovascular disease (CVD) events (N = 3903 participants). Incident CVD included fatal and nonfatal coronary heart disease, stroke, heart failure, and other CVD deaths. Cohort-stratified cause-specific hazard models for incident CVD and standard proportional hazard models for all-cause mortality were applied and included sex, age (stratified), BMI (<18.5, 18.5–23.9, 24–27.9, or 28 kg/m^2^), daily fat intake, daily caloric intake, risk of hypertension, daily saturated fat intake, geographic location (north or south), residence (urban or rural), and smoking status (never smoked or smoked). The dashed line indicates the cutoff for the 95th percentile of consumption (698.6 mg/d).

## 4. Discussion

In this prospective cohort study in China, 3903 adults aged 40 years or older were followed-up for a median of 14 years. Our analysis showcases that dietary cholesterol sources varied among subgroups, that total dietary cholesterol intake was significantly associated with the risk of incident CVD, and that cholesterol from poultry, seafood, and eggs sources were significantly associated with a reduced risk of incident CVD.

This is the first prospective cohort study to analyze the relationship between animal cholesterol sources and CVD in a Chinese population. Most current studies have focused on total cholesterol intake and CVD risk. Therefore, further large-scale studies are needed to confirm or refute these findings. The relationship between total cholesterol intake and CVD risk has been inconsistent in previous prospective cohort studies. Several large cohort studies have shown that higher dietary cholesterol intake is associated with an increased risk of CVD. This is consistent with our conclusions. A study conducted on postmenopausal women in the United States found that higher dietary cholesterol and protein intake were associated with increased risk of CVD and all-cause mortality [11]. The Swedish Mammography Cohort Study found that a higher intake of cholesterol (median, highest quintile 302 mg/d) was linked to a greater risk of total and ischemic stroke (20% and 29%, respectively) [12]. The results from a study of 29,615 participants six 6 cohorts with 5400 incident CVD events showed a significant association between dietary cholesterol intake and the risk of incident CVD and all-cause mortality in a dose–response relationship [8]. On the other hand, vegans may have significantly lower dietary cholesterol than the average omnivore. A cross-sectional study of 207 Spanish adults, categorizing participants as lacto-ovo-vegetarians (LOV), vegans (VEG), and omnivores (OMN), found that serum total cholesterol and LDL cholesterol were significantly lower in the LOV and VEG groups than in the OMN group (both *p* < 0.001) [30]. Similarly, a meta-analysis of 30 trials showed that plant-based diets lowered total cholesterol, LDL cholesterol, and apoB levels compared with the omnivore group, and the effect sizes were similar across age, continent, study duration, health status, intervention diet, intervention program, and study design [31].

In contrast, several studies have found no significant correlation between dietary cholesterol and stroke, incident coronary heart disease, or death from coronary heart disease, regardless of the method used for dietary assessment [13,32,33,34]. Moreover, a subset of cohort studies found no significant association between dietary cholesterol and fatal or nonfatal coronary heart disease or stroke when energy intake was used as a covariate in statistical models [35,36]. A prospective cohort study conducted with 1852 men with no prior history of venous thromboembolism or coronary heart disease found no evidence of a correlation between dietary cholesterol intake and venous thromboembolism or coronary heart disease (CHD) [37]. Similarly, a prospective cohort study of 2682 middle-aged and elderly men in eastern Finland (the Kuopio Ischaemic Heart Disease Risk Factor Study (KIHD)) found no evidence of an association between dietary cholesterol intake and venous thromboembolism, and this study found no statistically significant correlation between egg or cholesterol intake and stroke risk [38]. However, it is important to note that these findings are not conclusive, and further research is needed to fully understand the relationship between cholesterol intake and cardiovascular health.

Previous studies in Chinese populations have investigated the association between egg intake, egg cholesterol, lipids, and cardiovascular disease. Some studies have found a dose–response relationship between egg consumption and the incidence of cardiovascular disease, with a U-shaped curve [39,40]. For example, a prospective cohort study with a sample size of 20,688 individuals found that egg consumption was significantly associated with a lower risk of cardiovascular disease incidence [40]. A prospective cohort study of 500,000 individuals found that regular egg consumption was independently associated with an increased risk of CVD, ischemic heart disease (IHD), major coronary events (MCE), hemorrhagic stroke, and ischemic stroke. However, individuals who consumed eggs daily (up to <1 egg/day) had a 26% lower risk of hemorrhagic stroke [41]. It is important to note that these associations were observed after adjusting for potential confounders. Previous studies have produced conflicting results. The present study provides significant evidence on the relationship between total dietary cholesterol, cholesterol from different sources, and cardiovascular disease.

Based on the CNHS 2010–2012 study, Chinese older adults consumed an average of 217.4 mg of cholesterol per day. The study revealed that urban residents and men had higher levels of dietary cholesterol intake. Furthermore, the study found a positive correlation between dietary cholesterol intake and body mass index (BMI). Eggs, red meat, and seafood were identified as the primary sources of dietary cholesterol [23]. Our study’s findings are comparable to those of the previous study. However, it is important to note that the previous study was conducted with an older cohort of individuals over 60 years of age, so the distribution of baseline data may differ due to differences in study populations. According to the U.S. National Health and Nutrition Examination Survey (NHANES) cohort study conducted in the United States in 2013–2014, the average dietary cholesterol intake for U.S. adults was 293 mg/day (348 mg/day for men and 242 mg/day for women). Meat, eggs, grain products, and dairy products are the four food sources with the highest cholesterol intake. And the Mexican Americans had the highest cholesterol intake and density, while non-Hispanic Whites had the lowest [10]. The daily cholesterol intake may be higher in the United States or Europe compared to the Chinese population. Applying previous results from the U.S. or European cohorts to the Chinese population may be problematic.

Physiological studies have shown that cholesterol homeostasis is maintained through intracellular biosynthesis, biliary excretion, dietary cholesterol absorption, and blood cholesterol transport [42]. Hypercholesterolemia induced by genetics and/or diet is a major risk factor for atherosclerotic cardiovascular disease, as evidenced by the clear link between hypercholesterolemia and the formation of pathologic macrophage foam cells within the arterial wall [43]. Cholesterol biosynthesis accounts for 70–80% of daily cholesterol production. When dietary cholesterol intake is around 300 mg/d, human cholesterol biosynthesis produces approximately 800–1000 mg/d. Experimental studies have shown that the effect of dietary cholesterol on LDL-C and HDL-C is influenced by baseline cholesterol intake. Gender may also affect HDL-C response. Furthermore, dietary fatty acid intake has been found to impact changes in LDL-C and HDL-C [34]. Changes in dietary cholesterol were found to be positively associated with changes in LDL cholesterol concentrations [44]. Additional studies have shown that the effect of dietary cholesterol on serum cholesterol levels varies depending on whether an individual’s cholesterol synthesis is stimulated or down-regulated by an increase in dietary cholesterol intake. However, the extent to which these phenomena occur varies from person to person [45]. To understand the overall findings, it is important to differentiate individual health status and cholesterol sensitivity, dietary patterns, region, and ethnicity. Additionally, a moderate intake of high-cholesterol foods, such as eggs, was found to have no substantial effect on cholesterol homeostasis in healthy individuals [46].

Previous studies have shown a greater risk associated with animal proteins from red meat or processed meats compared to poultry, fish, and nuts, which have a lower risk of coronary heart disease. In contrast, there have been other studies showing no association between red meat and coronary heart disease [47,48,49]. This is similar to our findings. In a study of over 80,000 women, the consumption of red meat was found to be associated with an increased risk of CHD. However, the effects of nut, poultry, fish, and milk protein intake were different, as they were found to reduce CHD risk in the same subjects. Specifically, the consumption of nuts (1 serving/day) was found to reduce CHD risk by 30% compared to the consumption of red meat (1 serving/day). The study found that consuming low-fat dairy products was associated with a 13% reduction in CHD risk, while poultry and fish were associated with a 19% and 24% reduction, respectively, compared to consuming red meat (1 serving/day for all) [50]. The European Prospective Investigation into Cancer and Nutrition (EPIC) cohort found that only processed meats increase the risk of CVD and all-cause mortality, not red meat or poultry [51]. Fat-rich fish is a source of omega-3 polyunsaturated fatty acids, including eicosatetraenoic acid (EPA) and docosahexaenoic acid (DHA). Japan has the highest fish intake and the lowest risk of coronary heart disease in the world. In the Diet and Reinfarction Trial, men who were randomly assigned to increase their fish intake after a myocardial infarction experienced a 29% reduction in total mortality and a 32% reduction in coronary heart disease mortality compared to those who increased their grain intake or reduced their total fat intake to 30% [52]. In the typical Western diet, eggs are the primary source of dietary cholesterol. One egg yolk contains approximately 200 mg of cholesterol. According to studies, the consumption of eggs and egg products accounts for a quarter of the daily cholesterol intake of children and adults in the United States [53,54]. Saturated fat is known to strongly increase serum cholesterol, and eggs, which are relatively low in saturated fat, account for only 2.5% of the total saturated fatty acid intake of U.S. adults [54]. Our findings are similar, so higher egg-derived cholesterol intake may represent a lower saturated fatty acid intake and thus a lower risk of cardiovascular events.

We investigated the relationship between cholesterol intake from animal sources and incident CVD in Chinese individuals aged 40 years and older. We analyzed baseline cholesterol intake data from the CHNS cohort between 1991 and 2012 and found a significant association of total cholesterol intake and different sources of cholesterol with the development of CVD. However, this study still requires some issues to be addressed. As China has undergone significant economic and societal changes over the past three decades, we included the time of participants’ enrollment in the CHNS, a dynamic cohort, as a covariate in the model to account for changes in dietary levels. The dietary data in the CHNS cohort were obtained from dietary recalls on three consecutive days. However, this method may not always accurately predict long-term outcomes. To enhance the accuracy of the results, this study excluded 5% outliers. Additionally, due to the early design of the CHNS study questionnaire, the etiologic breakdown of CVD did not have sufficiently accurate questionnaire entries. Therefore, only CVD composite events were examined in this study. This study was unable to account for participants’ physical activity due to missing data in more than half of the baseline records. This may have introduced some bias into the results. The present study excluded participants’ self-reported diagnosed or undiagnosed hypertension at baseline. However, 25% of participants still had a mean systolic BP of more than 140 mm/Hg or a diastolic BP of more than 90 mm/Hg at the time of their BP measurements. We adjusted for the covariate of BP to increase the accuracy of the results. Possible randomization errors in the dietary review records may have weakened the true associations. Further research is needed for subsequent studies to account for the effect of preparation method on nutrients. There was also possible bias due to the observational design.

## 5. Conclusions

In conclusion, among Chinese adults aged 40 years and older, the risk of CVD was significantly associated with total dietary cholesterol intake, whereas cholesterol from poultry, seafood, and eggs was associated with a lower risk of CVD. As the first study to examine dietary sources of cholesterol and incident CVD in a Chinese population, the present study fills a gap in this area to some extent. In addition, this study summarized the dietary cholesterol composition of the Chinese population over 40 years of age. This finding will help future studies to make progress in refining the risk factors for CVD events, and we will also further explore the effects of dietary cholesterol changes on CVD events based on this study.

## Figures and Tables

**Table 1:** Baseline characteristics of CHNS participants over 40 categorized according to quartiles of cholesterol intake (N = 3039).

Characteristic	Q1 (0–79.0) (mg/d)	Q2 (79.0–183.3) (mg/d)	Q3 (183.3–362.5) (mg/d)	Q4 (362.5+) (mg/d)	*p* Value
N	976	975	976	976	
Age (years)	53.00 (45.00–63.00)	52.00 (45.00–62.00)	52.00 (45.00–62.00)	53.00 (46.00–62.00)	0.273
Calorie intake (kcal/d)	2302.93 (1859.83–2768.64)	2324.01 (1901.29–2811.87)	2263.85 (1861.39–2677.74)	2348.70 (1916.19–2760.74)	0.05
Poultry (mg/d) *	0.00 (0.00–0.00)	0.00 (0.00–0.00)	0.00 (0.00–0.00)	0.00 (0.00–1.47)	<0.001
Eggs (mg/d) *	0.00 (0.00–0.00)	0.00 (0.00–97.50)	168.08 (48.75–226.67)	390.00 (283.33–565.42)	<0.001
Red meats (mg/d) *	35.67 (17.83–54.94)	76.67 (27.00–107.00)	66.67 (23.00–123.05)	76.67 (30.67–131.08)	<0.001
Seafood(mg/d) *	0.00 (0.00–0.00)	0.00 (0.00–0.00)	0.00 (0.00–32.00)	0.00 (0.00–57.79)	<0.001
Smoking status, %					0.005
Never	627 (64.24%)	587 (60.21%)	633 (64.86%)	663 (67.93%)	
Smoke	349 (35.76%)	388 (39.79%)	343 (35.14%)	313 (32.07%)	
BMI, %					<0.001
<18.50	127 (13.01%)	90 (9.23%)	69 (7.07%)	48 (4.92%)	
18.50–23.99	645 (66.09%)	597 (61.23%)	554 (56.76%)	506 (51.84%)	
24.00–27.99	167 (17.11%)	213 (21.85%)	260 (26.64%)	322 (32.99%)	
28.00+	37 (3.79%)	75 (7.69%)	93 (9.53%)	100 (10.25%)	
Sex, %					0.008
Male	421 (43.14%)	478 (49.03%)	485 (49.69%)	484 (49.59%)	
Female	555 (56.86%)	497 (50.97%)	491 (50.31%)	492 (50.41%)	
Hypertension, %					<0.001
No risk of hypertension	775 (79.41%)	737 (75.59%)	725 (74.28%)	665 (68.14%)	
Risk of hypertension	201 (20.59%)	238 (24.41%)	251 (25.72%)	311 (31.86%)	
Residence, %					<0.001
Urban Site	267 (27.36%)	369 (37.85%)	499 (51.13%)	634 (64.96%)	
Rural Site	709 (72.64%)	606 (62.15%)	477 (48.87%)	342 (35.04%)	
Geographic location, %					<0.001
North China	320 (32.79%)	314 (32.21%)	408 (41.80%)	518 (53.07%)	
South China	656 (67.21%)	661 (67.79%)	568 (58.20%)	458 (46.93%)	
CVD, %					0.039
No CVD	874 (89.55%)	842 (86.36%)	851 (87.19%)	833 (85.35%)	
CVD	102 (10.45%)	133 (13.64%)	125 (12.81%)	143 (14.65%)	
Enrollment time					<0.001
Before 1999	841 (86.17%)	765 (78.46%)	640 (65.57%)	570 (58.40%)	
After 1999	135 (13.83%)	210 (21.54%)	336 (34.43%)	406 (41.60%)	
Alcohol consumption					0.15
Never	621 (64.35%)	589 (61.10%)	585 (60.31%)	578 (59.65%)	
Drink	344 (35.65%)	375 (38.90%)	385 (39.69%)	391 (40.35%)	

Data are presented as median (interquartile range) if normality was not met; *p*-values were obtained using Kruskal–Wallis test for the remaining quantitative variables and chi-squared test for categorical variables. Abbreviations: cardiovascular disease (CVD). * Intake of cholesterol from food sources.

## Data Availability

Restrictions apply to the availability of these data. Data were obtained from The China Health and Nutrition Survey (CHNS) and are available at https://www.cpc.unc.edu/projects/china (accessed on 16 January 2024) with the permission of The China Health and Nutrition Survey (CHNS).
